# Supplementary material for: Efficacy of aerial forward-looking infrared surveys for detecting polar bear maternal dens
Source: PLoS One. 2020 Feb 27;15(2):e0222744. doi: 10.1371/journal.pone.0222744 (PMC7046283; doi:10.1371/journal.pone.0222744)
Supplement: Attachment — Submitted filename: Response to reviewers of manuscript PONE.docx [file pone.0222744.s001.docx]

Response to reviewers of manuscript PONE-D-19-24951 entitled “Efficacy of aerial forward-looking infrared surveys for detecting polar bear maternal dens”

As corresponding author, I have responded to all requests below with my initials TSS followed by my response in red.

1. In your Methods section, please provide additional information regarding the permits you obtained for the work. Please ensure you have included the full name of the authority that approved the field site access and, if no permits were required, a brief statement explaining why.

TSS – I’ve added the following at the end of the METHODS section which reads as follows:

“This work was conducted under Federal Fish and Wildlife Permit #MA225854-1, issued by the US Fish and Wildlife Service, Division of Management Authority, Washington, D.C.”
Reviewer's Responses to Questions
**Comments to the Author**
1. Is the manuscript technically sound, and do the data support the conclusions?
The manuscript must describe a technically sound piece of scientific research with data that supports the conclusions. Experiments must have been conducted rigorously, with appropriate controls, replication, and sample sizes. The conclusions must be drawn appropriately based on the data presented.
Reviewer #1: Partly
Reviewer #2: Yes

2. Has the statistical analysis been performed appropriately and rigorously?

Reviewer #1: No

Reviewer #2: Yes

3. Have the authors made all data underlying the findings in their manuscript fully available?

The [PLOS Data policy](http://www.plosone.org/static/policies.action#sharing) requires authors to make all data underlying the findings described in their manuscript fully available without restriction, with rare exception (please refer to the Data Availability Statement in the manuscript PDF file). The data should be provided as part of the manuscript or its supporting information, or deposited to a public repository. For example, in addition to summary statistics, the data points behind means, medians and variance measures should be available. If there are restrictions on publicly sharing data—e.g. participant privacy or use of data from a third party—those must be specified.

Reviewer #1: Yes

Reviewer #2: No

4. Is the manuscript presented in an intelligible fashion and written in Standard English?

PLOS ONE does not copyedit accepted manuscripts, so the language in submitted articles must be clear, correct, and unambiguous. Any typographical or grammatical errors should be corrected at revision, so please note any specific errors here.

Reviewer #1: Yes

Reviewer #2: Yes

5. Review Comments to the Author

Please use the space provided to explain your answers to the questions above. You may also include additional comments for the author, including concerns about dual publication, research ethics, or publication ethics. (Please upload your review as an attachment if it exceeds 20,000 characters)

Reviewer #1: Overall, I think this research is valuable and draws important conclusions, but there is a disjoint throughout that hampers the clarity of the main message. There are details of some sections that seems to be lacking, or details that seem to be added unnecessarily. More structure, continuity across sections, and attention to those details could make the interpretation of the research much clearer. I also suggest some more statistical analysis of the collected data to support the conclusions (described in more detail below).

Suggested Edits

Line 40-42 – The in-text citation should not be a URL. That should be in the Reference section.

TSS – This has been moved to the Reference section as requested.

Line 44 – Move “therefore” to the beginning of the sentence to increase flow.

TSS - Moved and reworded for clarity.

Line 59-61 – You should add that you looked at weather and snow depth in your objective (or even just environmental variables), to set up the specific direction of the study early on.

TSS – I’ve reworded this as suggested. It now reads “The purpose of this study was to evaluate the influence of environmental variables (e.g., snow depth, wind, relative humidity, etc.) on the efficacy of industry-operated aerial FLIR surveys (hereafter referred to as “industry AFS”) and to make recommendations for future den detection and avoidance efforts.”

Line 72 – Figure 1- different symbology is used for some municipalities, so I suggest a legend denoting what each means.

TSS – A legend has been added differentiating between towns/villages, oil facilities, and polar bear den sites.

Line 88-89 – This sentence is redundant as it is restating what was said in the prior sentence.

TSS – Yes, that was redundant, so it has been deleted.

Line 91-92 – You are either missing a closing bracket, or you have an extra opening bracket before the URL.

TSS – Correct. I’ve closed the ( ) with the missing right bracket. Good catch! Four of us have missed that to date.

Line 103 – Is there a way you can explain what the “right” signature looks like? Or cite a description from another paper. The lack of description would make reproducibility difficult.

TSS – I’ve inserted a citation of York et al. (2004) because they thoroughly discuss what the properties of a hot spot include. They also recommend practicing with actual footage. I also added this verbiage “In general, den-associated hotspots were several meters in diameter and noticeably warmer than the surrounding terrain.”

Line 105-106 – This would be a good place to mention which years you were not given weather data for.

TSS – I’ve added the years (2004-2007) for which we obtained supplemental weather data.

Line 114 – Personnel instead of personal.

TSS – changed to Personnel

Methods – You talk about collecting weather data and snow depth data, but you don’t appear to do any further analyses with them. It would be interesting to include an analysis on these data and present any relationships between weather and den detection, or snow depth and den detection in the results section.

TSS – We did collect snow depth data but in spring (when dens were opened and abandoned) this was many months past when AFS were conducted and could not be meaningfully correlated/compared with survey results. One would need to know snow depth at the time of the survey and the only way to do that would be to open an active den, which is not possible. Here we simply mention that we collected these variables associated with den sites but these have nothing to do with this study. Perhaps I should remove the sentence as it has no direct relevance to this study and perhaps is misleading?

Line 132 – You mention monitoring den locations to confirm maternal dens in the methods, but you don’t directly speak to recording the timing of den break-out. If you present the results, you should mention the methodology to keep all aspects of your study clear to the reader.

TSS – For clarity, as suggested, I’ve added the following in Methods “We monitored maternal dens by direct observation and by placement of video cameras that recorded bear activity. In this manner we were able to ascertain the precise timing of den opening, duration of stay post-breakout, and when family groups abandoned dens [3, 17].”

Results – Presentation of weather via table is good but does not tell the reader very much about the effects weather may have had on accurate den detection. If there are any prevailing relationships between weather variables and misidentified den sites it would be useful to summarize them in the text.

TSS – I’ve highlighted in BOLD those values listed in the table that are outside of the range of FLIR detectability. Eight of the surveys were too windy (> 10 km/h) and two were marginal (9.7 km/h). I’ve added text which makes these points clearer. “Weather variable values highlighted in **bold** indicate conditions that diminish and/or preclude den detectability by FLIR [8, 9, 10].”

You collected snow depth, so I would also love to see some numbers on how snow depth affected industry AFS den detection. If you do this, I would also suggest adding a sentence about these results into your abstract.

TSS – As discussed above, snow depth data would only be useful if we were able to collect it at the time of survey, which is not possible.

Discussion – You frequently mention the results from other work on the effects of snow and weather on den detection, but you don’t speak to it with your own data. You have the data, so you should explore these relationships and present them alongside the previous work you are citing (ie. Robinson et al. 2014 and Amstrup et al. 2004). You told us the results of industry AFS are not sufficient for detection, but how do they compare to previous work in terms of weather, snow, etc.?

TSS – To speak to those data, I’ve added the following paragraph in Results: In Table 2, weather variables that are outside the optimal operational window for FLIR are highlighted in bold. Eight of thirteen flights were conducted with winds (> 10 km/h) that likely obscured thermal signatures of dens. During these flights, twenty polar bear dens were misidentified (false positives and/or false negatives). Two other flights, close to the wind speeds that block den detection, had four misidentified den sites (Table 2). One flight was conducted below the recommended temperature-dew point spread of 2.8 oC, as recommended by Amstrup et al. [8]. Flying surveys outside of the recommended weather windows prescribed by York et al. [8], Amstrup et al. [8], and Robinson et al. [10] diminishes chances of den detection. That, in conjunction with snow covering dens possibly being > 1 m (Robinson et al. [10]), may account for the detection rates reported in Table 1.

Line 154-163 – You present a possible explanation, then immediately strike it down. The information here is valuable, but could you frame it in a way that is just providing a reason to discount the idea that bears entered dens late, without presenting it as a possibility first.

TSS – I’ve reframed it as suggested. It was framed the way it was originally because I heard from several industry persons who offered this up as an explanation as to why AFS surveys were unable to identify dens that later appeared.

Line 164 – 169 – This would be a good place to compare or contrast the industry results to the results from Amstrup et al. 2004. Return focus to your results, rather than on the results from previous work.

TSS – I’ve revised this paragraph as follows: To test the ability of AFS to detect dens, Amstrup et al. [8] flew multiple FLIR surveys over 23 denning bears for which exact locations were known by radio-telemetry. Only 7 of the 23 dens (30%) were detected on every flight, and 4 dens (17%) were not detected, underscoring the importance of repetitive surveys. However, between the years 2004—2016, AFS were conducted without repetition as recommended by Amstrup et al. [8].Weather conditions (e.g., wind, precipitation, temperature-dew point spread) and conducting surveys in daylight, or too soon after snow fall or drifting caused by high wind, were identified as principal reasons for detection failure. Robinson et al. [10] advised against conducting surveys during daylight hours and Amstrup et al. [8] concluded the probability of detecting dens in sunlight was essentially zero. None of these AFS surveys were conducted with the sun above the horizon. Robinson et al. [10] also reported that when wind was > 10 km/h den detection with FLIR was increasingly unlikely. Of the industry AFS listed in Table 2, 42% (5 of 12) were conducted with winds < 10 km/h. However, of those five surveys, two were conducted with prevailing winds very close to the detectability cutoff (9.7 km/h). Hence, only 25% (*n* = 3) of industry AFS, for which we have data on ambient conditions, were performed under wind conditions that were conducive to den detection with FLIR.

Line 171 – Mention that industry AFS followed this recommendation and only did surveys when the sun was down.

TSS – I inserted this statement: None of these AFS surveys were conducted with the sun above the horizon.

Line 177 – Once you say 3 surveys were done under the correct wind conditions, you should briefly summarize if those 3 had accurate detection.

TSS – I’ve added this summary text: However, those surveys included seven misidentified den sites (Table 2), which could have been due to factors other than wind (e.g., excessive snow depth over dens, objects generating false positives such as rocks, discarded barrels, exposed tundra).

Line 179 – Change “never detected” to “did not detect”.

TSS – rewritten as suggested.

Line 182 -190 – Again, in the methods you say you collected snow depth data, it would be very beneficial to include that in your analysis and results, and then discuss your results alongside the other publications you are discussing here. You mention “roof thickness that precludes detection by industry AFS is uncertain” but you have not included this in any results.

TSS – I have explained this above but will briefly restate here that those snow depth measurements were part of the many we recorded after bears abandoned their dens. However, snow depth is a very dynamic value and our measurements were made months after AFS flights were taken hence there is no expectation that our values would have been representative of the actual snow depth over dens at the time of survey. So, the desired comparison cannot be made – we don’t have the data.

Line 195 – Too many spaces after the period, make sure spacing is the same throughout.

TSS – removed.

Line 197 – Remove extra period after “dens”.

TSS – removed.

Line 207 – Change “essential to maximizing” to “essential for maximizing”.

TSS – changed to: “This makes clear that maximizing cub survival potential is essential for polar bears in this region to persist.”

Line 208-210 – Add “In regions” before “where” at the start of the sentence. i.e. In regions where…..

TSS – Added in.

Line 211 – Change to “… southern Beaufort Sea polar bear population requires.”

TSS – changed.

Line 215 – The location of the (Table 2) reference is misleading, table 2 only contains weather variables, placing it at the end of that list makes it seem like it should contain more.

TSS - Rewritten as follows; “The poor den detection rate of industry AFS is likely due to a combination of factors, including weather-related variables (e.g., wind, temperature-dew point spread, precipitation; Table 2), time of day, and den roof thickness.”

Line 224 – Remove comma between “surveys” and “is”.

TSS – Removed.

Line 232 – This is the first time you are mentioning helicopters are preferable to fixed wings. This should be included earlier and explained to some extent why helicopters are preferable or cited here.

TSS – I’ve added in this explanatory statement at the end of the fourth paragraph in Results: “Finally, Amstrup et al. [8] recommended helicopters be used over fixed-wing aircraft since they can hover, thus allowing a better look at thermal features from a variety of angles and for an extended period of time.”

Line 241 – “the answer” feels too definitive. Change to “a viable solution”

TSS – So changed.

Line 248 – “J. Wilder” is listed twice.

TSS – removed.

Reviewer #2: This is a fairly straight-forward study that addresses a wildlife management technique of importance in parts of the range of polar bears. Forward looking infrared imagery is a technique used to detect polar bear maternity dens and this study provides a quantitative assessment of the method. The study is well-designed, the data and analyses are appropriate, and it is well written. I have few comments but a few that may improve the final version. Given recent interest in opening more the of the North Slope of Alaska to hydrocarbon exploration/extraction, this is a timely contribution to the literature. The conclusion section could be shortened by a good bit without loss of information.

Detailed issues

20 – I like to see binomial names at first mention - same issue on line 34

TSS – fixed in both places.

26-7 – “previously recommended” – this can be deleted as they aren’t articulated in the abstract so “protocols” is sufficient

TSS – deleted as suggested.

29 – “hot spots” has an ecological / biological connotation (e.g., hot spots of biodiversity) so I would reword. I am aware of one denning paper that uses hot spot analyses (spatial ecological method) to identify den areas. Maybe “false positive” or something like that?

TSS – changed as recommended

34 – a reference on denning ecology would be useful here.

TSS – Done.

36 – some background for non-polar bear experts – “abandon dens” – where do they go?

TSS – I’ve added the following for clarity: Cubs are born in midwinter [1], and family groups abandon dens by mid-April and head to areas where seal hunting can resume [2, 3].

39 – use of “even” could be seen as a bias by some. It can be dropped without loss of content.

TSS – Good point. It has been deleted to avoid the appearance of bias.

43 – the temporal context of “increasing” would help put the study in context for non-experts

TSS – I have added text to put the increase in land denning into temporal context: “Simultaneously, the proportion of maternal polar bears choosing to den on land has been increasing for the past 2 decades [1, 5].”
45 – the “altricial” issue is stated in line 34 – once is enough. Maybe drop it from line 34. I don’t know the difference between “very altricial” and “altricial” – consider dropping “very”.

TSS – to avoid repetition I’ve deleted that and it now reads “Because polar bear cubs cannot leave the shelter of the den until approximately 3 months of age [6], disruption….”
51 – “Previous research” – requires a citation in this sentence not the following one.
TSS – I’ve moved those citations to the end of this sentence as suggested.
86 – when you give a year range, it is by default a “period” so it’s redundant – brevity is always welcome

TSS – Now reads “We assessed the efficacy of industry AFS for polar bear den site detection by comparing AFS data from 2004-2016 with ground-truth data we collected….”

109-110 – an explanation of dew point is not useful but you could explain why it is being measured (i.e., what effect it may have on the FLIR detection of dens)
TSS – I simply deleted that definition. The sentence now reads “Dew point incorporates the effect of pressure and temperature on relative humidity.”

140 – I’m not sure what “accurately” applies to here. It suggests that some dens were inaccurately detected. Can you drop this word? I’m thinking issues of accuracy and precision so the use of the word isn’t clear.

TSS – Agreed. So I’ve dropped the word.
Conclusions
Some of the wording could be tempered a bit. Use of words like “imperiled” (line 211) could be replaces with a more neutral term such as “threatened” (the US classification). Overall, this section could be shortened or remove the “Conclusions” header. A conclusion section shouldn’t equal in length the main discussion (at least in my opinion). I find lines 213-220 largely repetitive with the discussion.

TSS – I went through and changed words as suggested:

- Catastrophic -> steep
- Imperiled -> threatened

220 – “FLIR will never” – that is a overly broad statement – increases in technology are a possible factor. The current methods and technology cannot detect all dens.
TSS – rewritten as “Given current limitations, FLIR is unlikely to assure that all maternal dens can be located and hence protected.”
225 – “are likely to decline” to “may decline” – Predicting weather events/conditions is a challenge for climate change models.

TSS – changed as requested.

225-7 – “other technologies” – this sentence doesn’t add significantly to the manuscript. This point is made again on line 237. Once is too much in my opinion.

TSS – Deleted as requested.

228-233 – if you could condense this section it would help as the factors listed weren’t directly tested in the study (i.e., they weren’t use as co-variates in a binomial analysis – detected / not detected). Such an analysis is not necessary (I doubt such an analysis would work based on the samples size).

TSS – I’ve deleted the points 1—7 and have left the reader to go to those papers which present in detail what those ‘best practices’ are.

241-2 – this is the 3rd reference to new technology

TSS – Having eliminated the previous two mentions, I will leave this as a closing statement.
